# The Response of Broiler Chickens to Dietary Soybean Meal Reduction with Glycine and Cysteine Inclusion at Marginal Sulfur Amino Acids (SAA) Deficiency

**DOI:** 10.3390/ani10091686

**Published:** 2020-09-18

**Authors:** Usman Elahi, Jing Wang, You-biao Ma, Shu-geng Wu, Guang-hai Qi, Hai-jun Zhang

**Affiliations:** Key Laboratory of Feed Biotechnology, Ministry of Agriculture and Rural Affairs, National Engineering Research Center of Biological Feed, Feed Research Institute, Chinese Academy of Agricultural Sciences, Beijing 100081, China; usmanelahi@gmx.de (U.E.); wangjing@caas.cn (J.W.); myb0514@126.com (Y.-b.M.); wushugeng@caas.cn (S.-g.W.); zhanghaijun@caas.cn (H.-j.Z.)

**Keywords:** broiler, cysteine, glycine, methionine, reduced protein

## Abstract

**Simple Summary:**

Climate change, resource shrinkage, and greenhouse gasses emission are becoming a major issue that could be confronted by using reducing protein levels in poultry diet. Reduced protein with amino acids supplementation improved the overall performance of broiler chickens. Reduced protein diets with glycine supplementation could be the potential solution to maintain the growth performance of the chicken, thus reducing feed cost and nitrogen excretion.

**Abstract:**

The responses of broiler chickens to dietary protein reduction were investigated in the presence of glycine and cysteine inclusion at the marginal deficiency of sulfur-containing amino acids. A total of 432 broiler chickens were allotted to six dietary treatments; SP1 is standard protein diet with 100% total sulfur amino acids (TSAA), SP2 is standard protein diet with 85% TSAA, RP is reduced protein diet without glycine and cysteine supplementation, RPC is reduced protein diet with cysteine supplementation at 0.1%, and RPG is reduced protein diet with 1% glycine supplementation, while RPGC is reduced protein diet with 0.1% cysteine and 1% glycine supplementation. In this study, 4.5% protein is reduced in diets—thus, 17.5% CP (crude protein) for starter phase and 15.5% CP for the grower phase. Reduced protein diets contained 85% TSAA. Broiler chickens fed standard protein diet SP2 had superior bodyweight (BW) (*p* ≤ 0.05) in the starter and grower phase, average daily gain (ADG) (*p* ≤ 0.05) in the starter and entire feeding period, average daily feed intake (ADFI) (*p* ≤ 0.05) in the starter phase, and better feed conversion ratio (FCR) (*p* ≤ 0.05) in the starter, grower and entire feeding period; however, RPGC showed higher ADG (*p* ≤ 0.05) in the grower phase, and ADFI (*p* ≤ 0.05) in the grower and entire feeding period. RPC and RPG diet improved BW (*p* ≤ 0.05), ADG (*p* ≤ 0.05), ADFI (*p* ≤ 0.05), and better FCR (*p* ≤ 0.05) in starter, grower, entire feeding period compared to RP. The RPGC group had higher BW (*p* ≤ 0.05), ADG (*p* ≤ 0.05), ADFI (*p* ≤ 0.05) and better FCR (*p* ≤ 0.05) compared to the RPC group. Blood biochemical parameters showed that Broiler chickens fed on the SP2 diet had higher levels of total protein (TP) (*p* ≤ 0.05), albumin (ALB) (*p* ≤ 0.05), creatinine (CRE) (*p* ≤ 0.05), and aspartate aminotransferase (AST) (*p* ≤ 0.05) and, lower level of uric acid (UA) (*p* ≤ 0.05), blood urea nitrogen (BUN) (*p* ≤ 0.05), glucose (GLU) (*p* ≤ 0.05), and alanine aminotransferase (ALT) (*p* ≤ 0.05) in the starter phase; however, higher level of TP (*p* ≤ 0.05), GLU (*p* ≤ 0.05), CRE (*p* ≤ 0.05), and AST (*p* ≤ 0.05), and lower level of ALB (*p* ≤ 0.05), UA (*p* ≤ 0.05), and ALT (*p* ≤ 0.05) in the grower phase; RPGC had higher level of TP (*p* ≤ 0.05), UA (*p* ≤ 0.05), GLU (*p* ≤ 0.05), ALT (*p* ≤ 0.05) and AST (*p* ≤ 0.05), and lower level of ALB (*p* ≤ 0.05), BUN (*p* ≤ 0.05), and CRE (*p* ≤ 0.05) in the starter phase; however, in grower phase, RPGC had higher level of TP (*p* ≤ 0.05), and ALB (*p* ≤ 0.05), and lower level of UA (*p* ≤ 0.05), CRE (*p* ≤ 0.05), ALT (*p* ≤ 0.05), and AST (*p* ≤ 0.05). Free amino acids profile showed that broiler fed on standard protein diet SP2 had reduced the methionine (*p* ≤ 0.05) concentration; RPC increased the concentrations of taurine (*p* ≤ 0.05), phosphoethanolamine (*p* ≤ 0.05), threonine (*p* ≤ 0.05), valine (*p* ≤ 0.05), isoleucine (*p* ≤ 0.05), phenylalanine (*p* ≤ 0.05), ornithine (*p* ≤ 0.05), and lysine (*p* ≤ 0.05) and reduced the citrulline (*p* ≤ 0.05) concentration; RPG increased the concentration of glutamate (*p* ≤ 0.05), glycine (*p* ≤ 0.05), cysteine (*p* ≤ 0.05), and arginine (*p* ≤ 0.05), and decreased the concentration of tyrosine (*p* ≤ 0.05); and RPGC increased the concentration of serine (*p* ≤ 0.05) and reduced the concentration of hydroxyproline (*p* ≤ 0.05). Serum metabolites analysis showed that reduced protein downregulated the 54 metabolites; however, glycine fortification up-regulated the Benzamide, Pro-Ser, N-Carbamylglutamate, D-gluconate, and Gamma-Glutamylcysteine. Carcass quality showed that SP2 decreased the abdominal fat percentage (*p* ≤ 0.05). Nitrogen digestibility was higher by the diet RP (*p* ≤ 0.05). This study demonstrated that protein content could be reduced up to 4.5% with 1% glycine and 0.1% cysteine fortification in diet, which has the potential to inhibit the adverse effect of reduced protein and attain the standard growth performance.

## 1. Introduction

The increasing demand for animal products and concomitant limitation of land for crops resulting in a shortage of protein-rich feedstuff increased crop prices and affected the affordability of food. These crops are also used in the animal feed industry. In poultry production, the feed cost accounts for 70–80% of the total cost. Generally, it is assumed that corn–soybean meal-based diets for broiler chickens contain adequate crude protein (CP) with limiting amino acid supplementation. Poultry nutritionists noticed that the high cost of protein ingredients and increasing pressure to reduce nitrogen emissions to the environment could be controlled using reduced protein diets. Nitrogen excretion is ineluctable because it is an integral component of feed due to metabolic processes leading to a form of substances appendant in urinary excretion. The nitrogen excretion is damaging our habitat, as excreted nitrogen is dispersing into the air, soil, and water. Reduced protein diets with amino acid supplementation could be the potential solution to maintain the growth performances of the chickens, reduce feed cost and nitrogen emission.

Glycine plays a special role in feeding reduced protein diets in poultry. Glycine is limiting amino acid after methionine, lysine, and threonine in a corn-soybean meal-based diet of broiler chickens [1]. Poultry can convert glycine into serine and vice versa; however, these conversions are not adequate to support maximal growth. Glycine is considered important for uric acid synthesis for nitrogenous waster excretion, the formation of blood haem, amino acid anabolism including synthesis of scleroproteins (collagen, keratin, elastin), gut mucin glycoproteins, metabolism of arginine, threonine, cysteine, and methionine [2]. Glycine and serine are incorporated in almost all body proteins. Collagen and elastin are amongst the proteins richest in glycine. Keratin, which is mainly present in feathers and claws, contains high proportions of both glycine and serine. Deficiency of glycine and serine in feed can cause low skin strength and impaired feather development [1]. Methionine and cysteine are sulfur amino acids and are involved in intricate metabolic processes. Cysteine is involved in the synthesis of keratin in feathers; however, cysteine deficiency reduces the nutrient deposition in the breast muscle because keratin synthesis is the priority [3]. Methionine participates in the body protein synthesis and is an ingredient of many body parts, including muscles, organs, and feathers [4]. However, methionine is also involved in the synthesis of the polyamines. Methionine donates its methyl group to biological processes that resulted in homocysteine (a sulfur-containing compound) by using cystathionine, methionine, and serine to jointly synthesize cysteine. The deficiency or excess of methionine or cysteine in its diet affect a chicken’s performance [5]. The deficiency of methionine leads to reduced growth performance, immune functioning, and increased abdominal fat proportions and feather pecking [6,7,8,9]. The methionine requirement can be fulfilled only by methionine; however, the cysteine requirement can also be satisfied with methionine. Reduced protein diets supplemented with free amino acids to increase the all essential amino acids levels failed to accomplish the growth performance of a standard crude protein diets [10,11]. A diet containing less than 20% crude protein reduced the growth and fed efficiency even when the essential amino acid requirement was fulfilled [12]. Reduced protein diets containing 2.03% total glycine + serine fed to male broiler chickens from 1–21 d of age failed to accomplish standard growth performance; however, glycine in a reduced protein diet is capable of reforming the poor performance of broiler chickens from 1–21 d of age [13].

Reduced protein concentrations are usually affiliated with DL-methionine inclusion; this could be because cysteine is not usually added; thus, the methionine and total sulfur amino acids (TSAA) ratio in diets increased on DL-methionine supplementation, irrespective of the proper requirement of cysteine [14]. Increased inclusion of methionine showed no improvement in growth performance; however, the inclusion of cysteine exceeding the standard level reduced glycine efficiency [14]. Diet containing 2.32% total glycine + serine with marginal level of TSAA (methionine and cysteine) improved feed/gain (F:G) in female broiler chickens [8]. It is preferable to maintain a higher level of glycine than recommended in National Research Council [15] in reduced protein diets [11,12,16,17].

The addition of glycine in diets containing 16% or 18% crude protein significantly improved the bodyweight at 21 d [18,19,20]. A diet containing 17% to 18% crude protein with an adequate level of glycine and serine resulted in standard growth and feed efficiency and is comparable to the diet containing more than 20% crude protein [12,16,21]. The addition of 0.2% or 0.4% glycine significantly improved overall performance, but the improvement was greatest in reduced protein diets [20].

The optimal level of glycine to support maximum growth and feed conversion in chickens is 1.76% to 1.8% (glycine + serine) for 7–20 d of age [16], 2.32% (glycine + serine) in 16% crude protein diet for female broiler chickens 0–17 d of age [12], and 2.08% (glycine + serine) in 19% crude protein for male broiler chickens 0–21 d of age [17].

It is hypothesized that a balance between methionine and cysteine with 0.1% cysteine and 1% glycine in a reduced protein diet may improve growth, blood biochemistry, plasma amino acids, serum metabolites, carcass, and nitrogen digestibility.

An experiment was conducted to investigate the effect of glycine addition and the balance between methionine and cysteine under the marginal deficiency of sulfur containing amino acids in a reduced protein diet on growth performance, blood biochemical parameters, serum metabolites, carcass quality, and nitrogen digestibility.

## 2. Materials and Methods

All experimental procedures were reviewed and approved by the Animal Care and Use Committee of the Feed Research Institute of the Chinese Academy of Agricultural Sciences (FRI-CAAS20181112).

### 2.1. Chickens and Diets

Four hundred and thirty–two, one–day–old Arbor Acres male broiler chickens (slow feathering) were weighed and randomly assigned to six treatments of six replicates containing 12 chickens each, reared on the floor with wood shavings as a bedding material. Each replicate size was 3 × 3 ft^2^. Broiler chickens had free access to feed and water and were maintained on a constant (23 h light: 1 h darkness) lightening program after the first three days with continuous light. The temperature in the chicken house was maintained to 33 °C for the first three days and then reduced by 3 °C each consecutive week until 28 °C. Six diets were formulated: SP1 is a standard protein diet contains the recommended CP, energy, and amino acids [15]; SP2 is also standard protein diet contains recommended CP and energy but 85% SAA; while four diets were formulated with 4.5% reduction in protein content—RP is reduced protein diet without any supplementation, RPC is reduced protein diet with 0.1% cysteine supplementation, RPG is reduced protein diet with 1% glycine supplementation, and RPGC is reduced protein diet with 1% glycine and 0.1% cysteine supplementation. All diets contained 1.12% true digestible Lys and equal concentrations of all essential amino acids (AA) by adding crystalline AA to meet the AA ratios. In this trial, 4.5% protein is reduced in diets; thus, 17.5% CP for the starter phase and 15.5% CP for the grower phase. The supplemented glycine in the reduced protein diets is 0.963% for the starter phase and 1.18% for the grower phase. Crystalline amino acids except lysine, methionine, threonine, and tryptophan used in the chicken diet were purchased from Hebei Guangrui Biological Products Co. Ltd. (Shijiazhuang, China). All diets were formulated to meet the AA requirements of broilers, which were determined by the AminoChick 2.0 (Evonik Industrial Group, Essen, Germany), a software tool for predicting optimum amino acids contents in poultry feed. The starter and grower dietary composition of standard and reduced protein diets are shown in Table 1, the nutritional level of standard and reduced protein diets are shown in Table 2, and the sulfur-containing amino acids and glycine addition levels in the reduced protein diets are shown in Table 3.

### 2.2. Growth Performance

Bodyweight (BW), feed offered (FO), and feed residue (FR) were documented for each pen on 0, 21, and 42 d of age. Mortality was documented daily for feed intake (FI) correction. Feed conversion ratio (FCR), average daily feed intake (ADFI), and average daily gain (ADG) were calculated using the documented data of BW, FO, FR, and mortality.

### 2.3. Blood Biochemical Parameters

One chicken was randomly selected from each replicate for blood sampling at 21 d and 42 d of age. Blood was sampled from the wing vein and kept in heparin-lithium-treated tubes. The blood was then centrifuged at 1800 g for 10 min, and the plasma was stored in a 1.5 mL Eppendorf tube (Eppendorf, Hamburg, Germany) at −30 °C until analysis. Total protein (TP), albumin (ALB), uric acid (UA), blood urea nitrogen (BUN), glucose (GLU), creatinine (CRE), alanine aminotransferase (ALT), and aspartate aminotransferase (AST) contents were determined with a biochemical analyzer (KHB-ZY 1280, Shanghai Kehua Bio-engineering Co., Ltd., Shanghai, China).

### 2.4. Plasma Amino Acid

One chicken was randomly selected from each replicate for blood sampling at 42 d of age. The blood was kept in heparin-lithium-treated tubes, then centrifuged at 1800 g for 10 min, and the plasma was stored in a 1.5 mL Eppendorf tube at −30 °C until analysis. Plasma amino acid concentrations were determined with an automatic amino acid analyzer (L-8800; Hitachi, Tokyo, Japan) with a ninhydrin reagent and lithium buffer system.

### 2.5. Metabolomics Analysis

One chicken was randomly selected (used for plasma amino acid analysis and carcass quality) from each replicate for blood sampling at 42 d of age. Blood samples were collected from the jugular vein and kept in serum tubes (Sarstedt, Nürnberg, Germany). Blood samples were kept on ice until centrifugation at 1811× *g* for 10 min. Serum aliquots were stored at −80 °C until analysis. Serum samples were thawed on ice and maintained at 4 °C throughout the analysis. Methanol was added at 3.5 volumes to each serum sample (250 µL), along with 700 ng of internal standard (3.5 µL deuterated tryptophan at a concentration of 200 ng/µL; OlChemim, s.r.o, Olomouc, Czech Republic), for a final volume of 1128.5 µL. Samples were then incubated for 12 h at −20 °C and then spun at 14,000× *g* for 15 min at 4 °C. Metabolites from the supernatants were harvested and filtered through a 0.2 µm, 5 mm PTFE disc syringe filters into vials. Samples were then sealed and subjected to the quantitative time of flight liquid chromatography tandem mass spectrometry (QTOF/MS) analyses. Liquid chromatography mas spectrometry (LC-MS) analyses were performed on an Agilent 6545 QTOF in positive ion mode with a dual AJS ESI system and an Agilent 1290 Infinity LC system (Agilent Technologies, Santa Clara, CA, USA). Briefly, 5 µL of each sample was separated on an Agilent C-18 Poroshell column using the following program: 0 min 2% B, 15 min 90% B, hold for 1 min, 17 min, 2% B, 32 min 2% B (buffer A: H2O 0.1% formic acid; buffer B: MeOH 0.1% formic acid). The mass spectrometer was run in MS2 scan mode, with a range of 50–1700 amu. Ion source indicators were as follows: gas temperature 250 °C, source gas flow 8 L/min., nebulizer 25 psi, sheath gas temperature 350 °C, sheath gas flow 10 L/min, capillary voltage 3500 V (positive ion mode), and nozzle voltage 500 V (positive ion mode). Features were selected using data-dependent acquisition, at a given time point were retained, used to generate a mass spectral profile, and then excluded for a 30-s window. Data were analyzed using the BMK cloud (https://international.biocloud.net). Features were identified, searched in the Kyoto Encyclopedia of Genes and Genomes (KEGG) metabolite library and visualized using the BMK cloud.

### 2.6. Carcass Quality

At 42 d of age, two chickens from each replicate were randomly selected, slaughtered, and defeathered. The carcass weight was calculated by removing the blood, feathers, head, feet, and visceral organs, except the lungs and kidneys. The carcass yield was expressed as a percentage of live weight. The wings, legs, breast meat, and abdominal fat were removed from the carcass and individually weighted and expressed as the ratio to the eviscerated carcass weight. Grilling chicken wings are favorite food to some consumers, so the weight of the wings can partly stand for the edible part of the carcass.

### 2.7. Fecal Collection and Nitrogen Digestibility

Feces were collected from the selected replicates of each treatment for three consecutive days from 17 to 19 and 27 to 29 of the experiment. Broiler chickens were kept in metabolic cages for five days in each phase (two days for adaptation to the cages and three days for excreta collection). Diets were weighed and documented at the start and at the end of the fecal collection days. Samples were collected once a day at 15:00, weighed, and stored at −20 °C until further analysis. Fecal samples were thawed at 3 °C, weighed, and homogenized. Fecal samples were analyzed for dry matter (drying in the oven at 103 °C for 8 h) and nitrogen content (ID: 968.07 [22]). Nitrogen digestibility was calculated using the following formula:

$$\mathrm{Digestibility}\;\mathrm{coefficient}\;\mathrm{of}\;\mathrm{nitrogen}\;=\;\frac{(\mathrm{Feed}\;\mathrm{Intake}\;\times \;\mathrm{Nitrogen}\;\mathrm{content}\;\mathrm{in}\;\mathrm{Feed})\;-\;(\mathrm{Excretion}\;\times \;\mathrm{Nitrogen}\;\mathrm{content}\;\mathrm{in}\;\mathrm{Feces})\;\times \;100}{\mathrm{Feed}\;\mathrm{Intake}\;\times \;\mathrm{Nitrogen}\;\mathrm{Content}\;\mathrm{in}\;\mathrm{Feed}}$$

### 2.8. Statistical Analysis

Data were presented as mean ± standard error and were subjected to one-way ANOVA using the GLM procedure of IBM SPSS statistics for windows (version 25.0, IBM Corp. Armonk, NY, USA) after the assessment of normality and homoscedasticity. Data in the low CP groups were analyzed as a 2 × 2 factorial arrangement. For better presentation of the results, the specific probabilities between low CP groups were listed in the tables. Differences among treatments were determined by Tukey’s honestly significant difference (HSD) test at a 5% level of significance.

## 3. Results

### 3.1. Growth Performance

Results of growth performance during the trial are presented in Table 4. A total of 52 mortalities were recorded during the trial. Broiler chickens fed on the SP2 diet had significantly higher BW (*p* ≤ 0.05), ADG (*p* ≤ 0.05), and ADFI (*p* ≤ 0.05) in starter and grower phase and FCR (*p* ≤ 0.05) for grower, and entire feeding period compared to the broiler chickens fed SP1 diet. In comparison between SP2 and RPs showed that broiler chickens fed on the SP2 diet had higher BW (*p* ≤ 0.05) in the starter and grower phase, higher ADG (*p* ≤ 0.05) in the starter and entire feeding period, higher ADFI (*p* ≤ 0.05) in the starter phase, and better FCR (*p* ≤ 0.05) in the starter, grower and entire feeding period; however, RPGC (reduced protein diet with cysteine and glycine supplementation)showed higher ADG (*p* ≤ 0.05) in the grower phase, and higher ADFI (*p* ≤ 0.05) in the grower and entire feeding period. Cysteine supplementation in the RPC diet curbed the adverse effects of the reduced protein and significantly improved BW (*p* ≤ 0.05), ADG (*p* ≤ 0.05), ADFI (*p* ≤ 0.05), and better FCR (*p* ≤ 0.05) in starter, grower, and the entire feeding periods compared to the broiler chickens fed the RP diet. Glycine supplementation in the diet also abated the inimical effects of reduced protein. The RPG diet improved BW (*p* ≤ 0.05), ADG (*p* ≤ 0.05), ADFI (*p* ≤ 0.05), and resulted in better FCR (*p* ≤ 0.05) in starter, grower, and the entire feeding periods compared to the broiler chickens fed the RP diet. The RPGC diet enhanced the cysteine efficiency and had higher BW (*p* ≤ 0.05) in the grower phase; higher ADG (*p* ≤ 0.05) in starter, grower, and entire feeding periods; ADFI (*p* ≤ 0.05) in the starter and entire feeding periods; and better FCR (*p* ≤ 0.05) in the starter, grower, and the entire feeding periods compared to the broiler chickens fed on the RPC diet. Boiler chickens fed on the RPGC diet showed equivalent growth performance to the chickens fed on the SP2 diet except the FCR.

### 3.2. Blood Biochemical Parameters

The reduced protein diet affected the blood biochemical parameters (Table 5). Broiler chickens fed on the SP2 diet had higher levels of TP (*p* ≤ 0.05), ALB (*p* ≤ 0.05), CRE (*p* ≤ 0.05), and AST (*p* ≤ 0.05) and lower levels of UA (*p* ≤ 0.05), BUN (*p* ≤ 0.05), GLU (*p* ≤ 0.05), and ALT (*p* ≤ 0.05) in the starter phase; however, they had higher levels of TP (*p* ≤ 0.05), GLU (*p* ≤ 0.05), CRE (*p* ≤ 0.05), and AST (*p* ≤ 0.05) and lower levels of ALB (*p* ≤ 0.05), UA (*p* ≤ 0.05), and ALT (*p* ≤ 0.05) in the grower phase compared to the broiler chickens fed the SP1 diet. Comparison between SP2 and RPs showed no effect on blood biochemical parameters. Broiler chickens from the RPC group had higher levels of TP (*p* ≤ 0.05), and CRE (*p* ≤ 0.05) and lower levels of ALB (*p* ≤ 0.05), UA (*p* ≤ 0.05), BUN (*p* ≤ 0.05), GLU (*p* ≤ 0.05), and AST (*p* ≤ 0.05) in the starter phase; however, in the grower phase, RPC had higher levels of UA (*p* ≤ 0.05), CRE (*p* ≤ 0.05), and ALT (*p* ≤ 0.05) and lower levels of ALB (*p* ≤ 0.05), GLU (*p* ≤ 0.05), and AST(*p* ≤ 0.05) compared to the broiler chickens fed the RP diet. Comparison between RP and RPG showed that the RPG group had lower levels of TP (*p* ≤ 0.05), ALB (*p* ≤ 0.05), BUN (*p* ≤ 0.05), and GLU (*p* ≤ 0.05), and higher levels of UA (*p* ≤ 0.05), and ALT (*p* ≤ 0.05) in starter phase; however, in grower phase RPG had lower levels of TP (*p* ≤ 0.05), ALT (*p* ≤ 0.05), and AST (*p* ≤ 0.05) and higher levels of ALB (*p* ≤ 0.05), UA (*p* ≤ 0.05), GLU (*p* ≤ 0.05), and CRE (*p* ≤ 0.05) compared to the broiler chickens fed the RP diet. Broiler chickens fed on the RPGC diet had higher levels of TP (*p* ≤ 0.05), UA (*p* ≤ 0.05), GLU (*p* ≤ 0.05), ALT(*p* ≤ 0.05), and AST (*p* ≤ 0.05) and lower levels of ALB (*p* ≤ 0.05), BUN (*p* ≤ 0.05), and CRE (*p* ≤ 0.05) in the starter phase; however, in the grower phase, RPGC had higher levels of TP (*p* ≤ 0.05) and ALB (*p* ≤ 0.05) and lower levels of UA (*p* ≤ 0.05), CRE (*p* ≤ 0.05), ALT (*p* ≤ 0.05), and AST (*p* ≤ 0.05) compared to the RPC group.

### 3.3. Plasma Amino Acids

Blood free amino acids (Table 6, Table 7 and Table 8) showed that broiler chickens fed on the SP2 diet reduced the concentrations of phosphoserine (*p* ≤ 0.05), taurine (*p* ≤ 0.05), phosphoethanolamine (*p* ≤ 0.05), threonine (*p* ≤ 0.05), serine (*p* ≤ 0.05), asparagine (*p* ≤ 0.05), glutamate (*p* ≤ 0.05), glutamine (*p* ≤ 0.05), glycine (*p* ≤ 0.05), alanine (*p* ≤ 0.05), α aminobutyric acid (*p* ≤ 0.05), valine (*p* ≤ 0.05), cysteine (*p* ≤ 0.05), methionine (*p* ≤ 0.05), isoleucine (*p* ≤ 0.05), leucine (*p* ≤ 0.05), tyrosine (*p* ≤ 0.05), phenylalanine (*p* ≤ 0.05), histidine (*p* ≤ 0.05), ornithine (*p* ≤ 0.05), lysine (*p* ≤ 0.05), hydroxyproline (*p* ≤ 0.05), and proline (*p* ≤ 0.05) and increased the arginine (*p* ≤ 0.05) concentration compared to the broiler chickens fed the SP1 diet. In comparison between SP2 and RPs showed that SP2 reduced the methionine (*p* ≤ 0.05) concentration; RPC increased the concentrations of taurine (*p* ≤ 0.05), phosphoethanolamine (*p* ≤ 0.05), threonine (*p* ≤ 0.05), valine (*p* ≤ 0.05), isoleucine (*p* ≤ 0.05), phenylalanine (*p* ≤ 0.05), ornithine (*p* ≤ 0.05), and lysine (*p* ≤ 0.05) and reduced the citrulline (*p* ≤ 0.05) concentration; RPG increased the concentration of glutamate (*p* ≤ 0.05), glycine (*p* ≤ 0.05), cysteine (*p* ≤ 0.05), and arginine (*p* ≤ 0.05), and decreased the concentration of tyrosine (*p* ≤ 0.05); and RPGC increased the concentration of serine (*p* ≤ 0.05) and reduced the concentration of hydroxyproline (*p* ≤ 0.05). Cysteine supplementation in the RPC diet increased the concentrations of taurine (*p* ≤ 0.05), phosphoethanolamine (*p* ≤ 0.05), threonine (*p* ≤ 0.05), serine (*p* ≤ 0.05), asparagine (*p* ≤ 0.05), glutamate (*p* ≤ 0.05), glycine (*p* ≤ 0.05), valine (*p* ≤ 0.05), cysteine (*p* ≤ 0.05), isoleucine (*p* ≤ 0.05), leucine (*p* ≤ 0.05), phenylalanine (*p* ≤ 0.05), ornithine (*p* ≤ 0.05), arginine (*p* ≤ 0.05), and proline (*p* ≤ 0.05), and decreased the concentration of methionine (*p* ≤ 0.05), tyrosine (*p* ≤ 0.05), and hydroxyproline (*p* ≤ 0.05); however, glycine supplementation in the RPG diet increased the concentrations of urea (*p* ≤ 0.05), threonine (*p* ≤ 0.05), serine (*p* ≤ 0.05), glutamate (*p* ≤ 0.05), glycine (*p* ≤ 0.05), cysteine (*p* ≤ 0.05), 3-methylhistidine (*p* ≤ 0.05), ornithine (*p* ≤ 0.05), lysine (*p* ≤ 0.05), arginine (*p* ≤ 0.05), and proline (*p* ≤ 0.05) and decreased the concentrations of taurine (*p* ≤ 0.05), phosphoethanolamine (*p* ≤ 0.05), asparagine (*p* ≤ 0.05), valine (*p* ≤ 0.05), methionine (*p* ≤ 0.05), isoleucine (*p* ≤ 0.05), phenylalanine (*p* ≤ 0.05), and hydroxyproline (*p* ≤ 0.05) compared to the broiler chickens fed on the RP diet. Broiler chickens from the RPGC group increased the concentrations of serine (*p* ≤ 0.05), glycine (*p* ≤ 0.05), citrulline (*p* ≤ 0.05), and cysteine (*p* ≤ 0.05) and reduced the concentrations taurine (*p* ≤ 0.05), threonine (*p* ≤ 0.05), asparagine (*p* ≤ 0.05), glutamate (*p* ≤ 0.05), glutamine (*p* ≤ 0.05), methionine (*p* ≤ 0.05), isoleucine (*p* ≤ 0.05), leucine (*p* ≤ 0.05), tyrosine (*p* ≤ 0.05), phenylalanine (*p* ≤ 0.05), ornithine (*p* ≤ 0.05), lysine (*p* ≤ 0.05), arginine (*p* ≤ 0.05), and hydroxyproline (*p* ≤ 0.05) compared to the broiler chickens from the RPC group.

### 3.4. Metabolomics Analysis

The differential metabolites in the metabolic pathway were predicted using the KEGG orthology (KO) analysis and Kyoto Encyclopedia of Genes and Genomics (KEGG) pathways.

#### 3.4.1. SP1 vs. SP2

**Figure 1:**
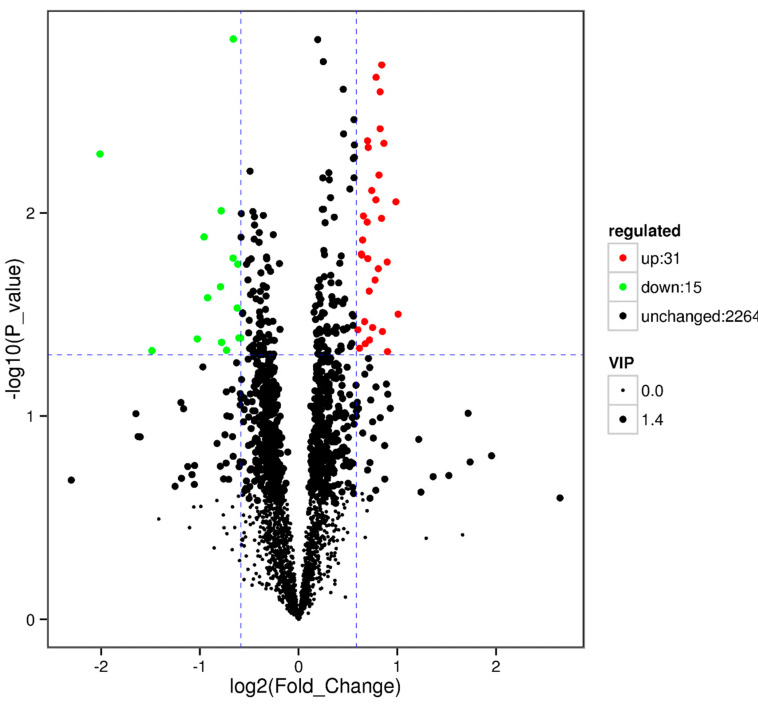
Volcano plot showing metabolomics data of SP2. The horizontal axis indicates the large magnitude fold-changes and the vertical axis indicates the high statistical significance (−log10 of *p* value). The dashed line shows where *p* = 0.05 with points above the line having *p* < 0.05 and points below the line having *p* > 0.05. Each point represents a metabolite, and the point size represents the VIP (variable importance in projection) value.

**Figure 2:**
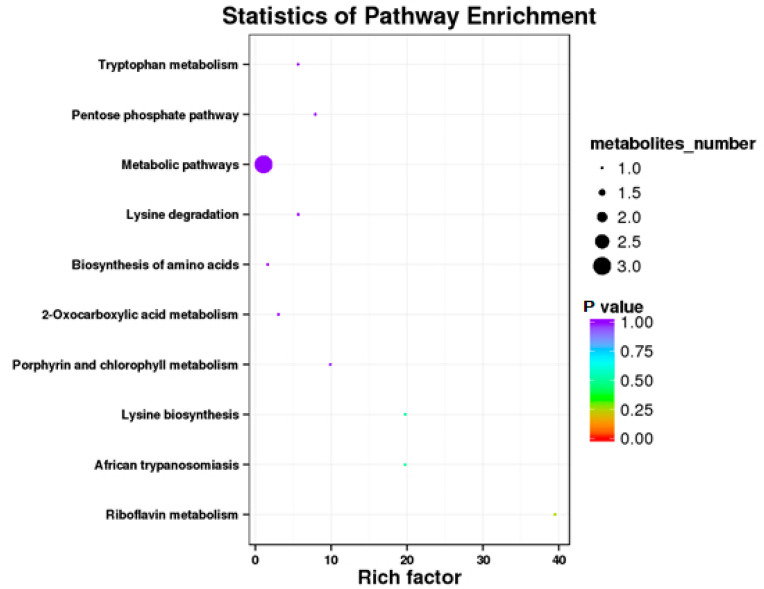
Kyoto Encyclopedia of Genes and Genomics orthology (KO) enrichment analysis of differential metabolite in response to SP2. The horizontal axis denoted the rich factor and the vertical axis indicated the pathway name. The right black histogram indicates the number of metabolites in each cluster. The larger the rich factor, the higher the degree of enrichment. The *p* value ranges from 0.00 to 1.00.

The results showed that some metabolic pathways in the SP2 group changed significantly as compared with the SP1. Figure 1 and Table 9 shows that 31 metabolites up-regulated, 15 down-regulated, and 2264 had no effect on the processes. The KEGG pathways, including the metabolic pathway, tryptophan metabolism, pentose phosphate pathway, lysine degradation, biosynthesis of amino acids, 2 oxocarboxylic acid metabolism, porphyrin and chlorophyll metabolism, lysine biosynthesis, African trypanosomiasis, and riboflavin metabolism, were found to be the top 10 annotated pathways for the SP2 (Figure 2).

#### 3.4.2. RP vs. RPC

**Figure 3:**
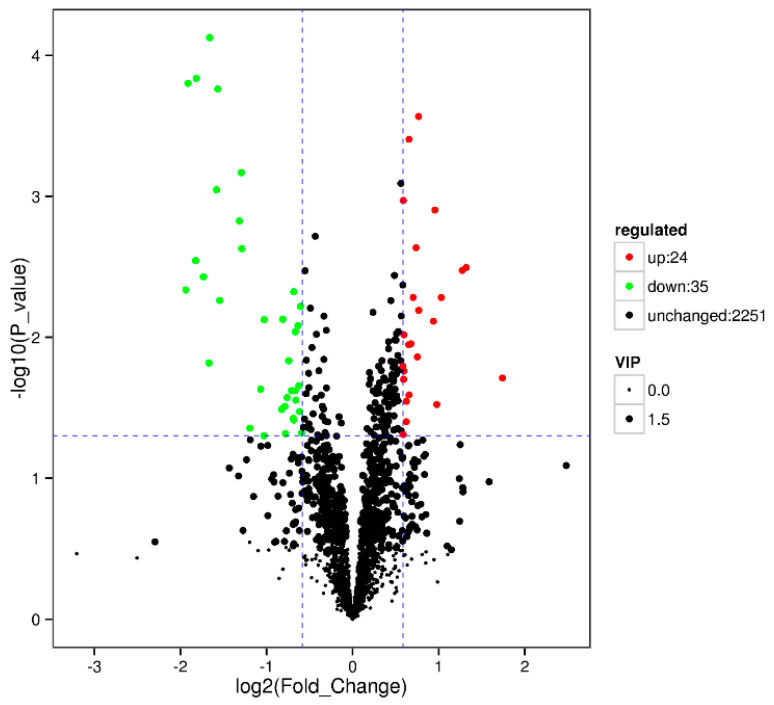
Volcano plot showing metabolomics data of RPC. The horizontal axis indicates the large magnitude fold-changes and the vertical axis indicates the high statistical significance (−log10 of *p* value). The dashed line shows where *p* = 0.05 with points above the line having *p* < 0.05 and points below the line having *p* > 0.05. Each point represents a metabolite, and the point size represents the VIP value.

**Figure 4:**
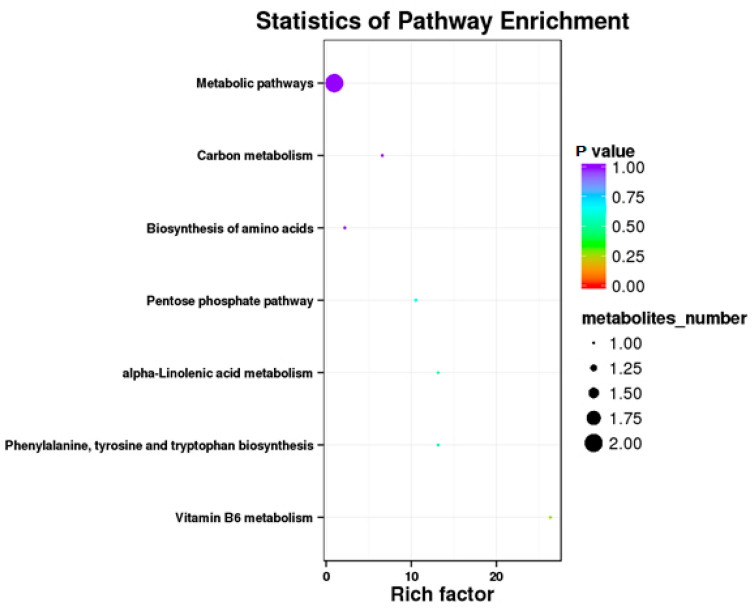
Kyoto Encyclopedia of Genes and Genomics orthology (KO) enrichment analysis of differential metabolite in response to RPC. The horizontal axis denoted the rich factor and the vertical axis indicated the pathway name. The right black histogram indicates the number of metabolites in each cluster. The larger the rich factor, the higher the degree of enrichment. The *p* value ranges from 0.00 to 1.00.

Results showed that some metabolic pathways in the RPC group changed significantly as compared with the RP. Figure 3 and Table 10 show that 24 metabolites up-regulated, 35 down-regulated, and 2251 had no effect on the processes. The KEGG pathways, including the metabolic pathway, carbon metabolism, biosynthesis of amino acids, pentose phosphate pathway, alpha lenolenic acid metabolism, phenlyalanine tyrosine and tryptophan biosynthesis, and vitamin B6 metabolism were found to be the annotated pathways for the RPC (Figure 4).

#### 3.4.3. RPC vs. RPGC

**Figure 5:**
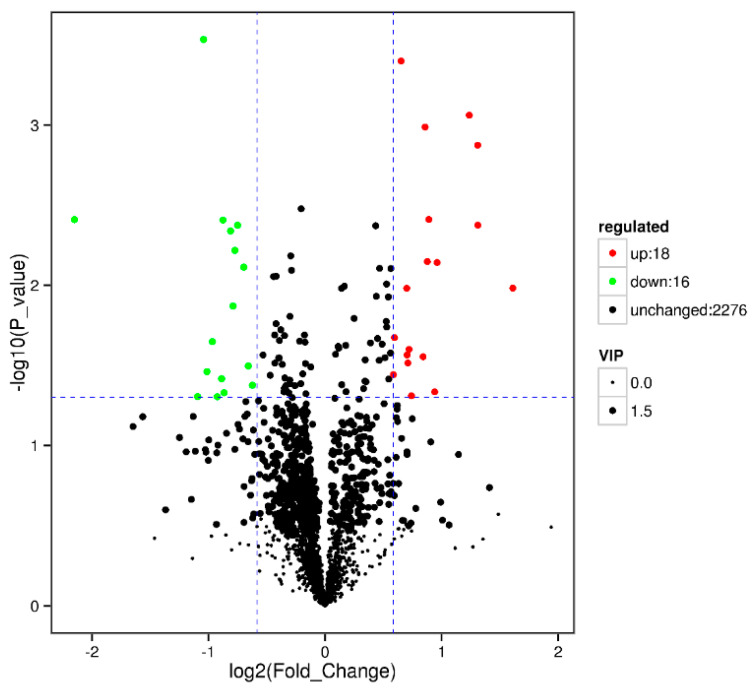
Volcano plot showing metabolomics data of RPGC. The horizontal axis indicates the large magnitude fold-changes and the vertical axis indicates the high statistical significance (−log10 of *p* value). The dashed line shows where *p* = 0.05 with points above the line having *p* < 0.05 and points below the line having *p* > 0.05. Each point represents a metabolite, and the point size represents the VIP value.

**Figure 6:**
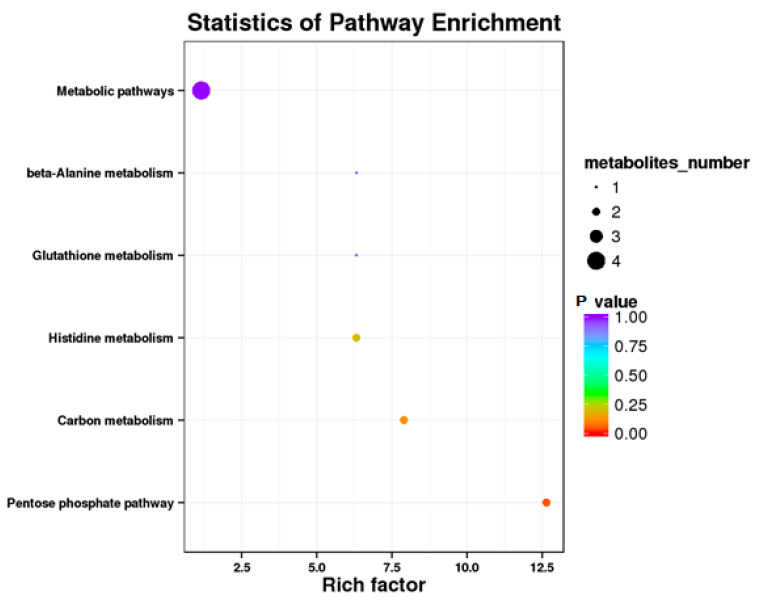
Kyoto Encyclopedia of Genes and Genomics orthology (KO) enrichment analysis of differential metabolite in response to RPGC. The horizontal axis denoted the rich factor and the vertical axis indicated the pathway name. The right black histogram indicates the number of metabolites in each cluster. The larger the rich factor, the higher the degree of enrichment. The *p* value ranges from 0.00 to 1.00.

Results showed that some metabolic pathways in the RPGC group changed significantly as compared with the RP. Figure 5 and Table 11 shows that 18 metabolites up-regulated, 16 down-regulated, and 2276 had no effect on the processes. The KEGG pathways, including the metabolic pathway, beta alanine metabolism, glutathione metabolism, histidine metabolism, carbon metabolism, and pentose phosphate pathway were found to be the annotated pathways for the RPGC (Figure 6).

#### 3.4.4. RPGC vs. SP2

**Figure 7:**
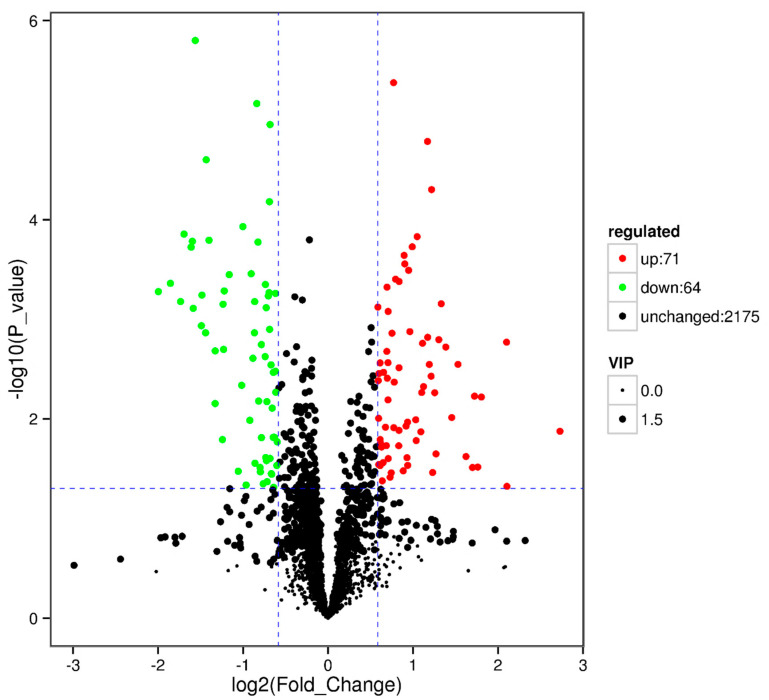
Volcano plot showing metabolomics data of RPGC. The horizontal axis indicates the large magnitude fold-changes and the vertical axis indicates the high statistical significance (−log10 of *p* value). The dashed line shows where *p* = 0.05 with points above the line having *p* < 0.05 and points below the line having *p* > 0.05. Each point represents a metabolite, and the point size represents the VIP value.

**Figure 8:**
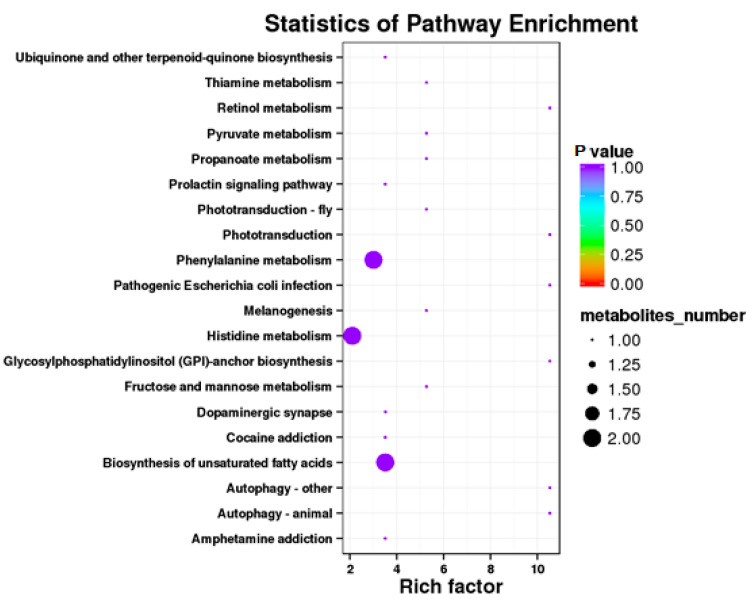
Kyoto Encyclopedia of Genes and Genomics orthology (KO) enrichment analysis of differential metabolite in response to RPGC. The horizontal axis denoted the rich factor and the vertical axis indicated the pathway name. The right black histogram indicates the number of metabolites in each cluster. The larger the rich factor, the higher the degree of enrichment. The *p* value ranges from 0.00 to 1.00.

Results showed that some metabolic pathways in the RPGC group changed significantly as compared with the SP2. Figure 7 and Table 12 shows that 71 metabolites up-regulated, 64 down-regulated, and 2175 unchanged the processes. The KEGG pathways, ubiquinone and other terpenoid quinone biosynthesis, thiamine metabolism, thiamine metabolism, retinol metabolism, pyruvate metabolism, propanoate metabolism, prolactin signaling pathway, phototransduction fly, phototransduction, phenylalanine metabolism, pathogenetic escherichia coli infection, melanogenesis, histidine metabolism, glycolsylphosphatidylinositol (GPI) anchor biosynthesis, fructose and mannose metabolism, dopaminergic synapse, cocaine addiction, biosynthesis of unsaturated fatty acids, autophagy other, autophagy animal, and amphetamine addiction were found to be the annotated pathways for the RPGC (Figure 8).

### 3.5. Carcass Quality

Standard or reduced protein diets with or without supplementation did not affect the carcass quality except abdominal fat percentage (Table 13). Broiler chickens fed on the SP2 diet increased the abdominal fat percentage (*p* ≤ 0.05) compared to the broiler chickens fed on the SP1 diet. Comparison between SP2 and RPs showed that SP2 decreased the abdominal fat percentage (*p* ≤ 0.05). Cysteine supplementation in the RPC diet increased the abdominal fat percentage (*p* ≤ 0.05); however, glycine supplementation in the RPG diet reduced the abdominal fat percentage (*p* ≤ 0.05) compared to the RP diet. Broiler chickens from the RPGC group increased the abdominal fat percentage (*p* ≤ 0.05) compared to the chickens from RPC group.

### 3.6. Nitrogen Digestibility

Results during the trial are presented in Table 14. Broiler chickens fed on SP2 diet reduced the nitrogen digestibility (*p* ≤ 0.05) in the grower phase compared to broiler chickens fed on SP1 diet. Comparison between SP2 and RPs showed that broiler chickens fed on RPC diet increased nitrogen digestibility (*p* ≤ 0.05) for the starter phase; however, SP2 reduced the nitrogen digestibility (*p* ≤ 0.05) for the grower phase. Cysteine supplementation in the RPC diet reduced the nitrogen digestibility (*p* ≤ 0.05) in the grower phase; however, glycine supplementation in the RPG diet also reduced the nitrogen digestibility (*p* ≤ 0.05) in the grower phase compared to the RP group. The RPGC group reduced the nitrogen digestibility (*p* ≤ 0.05) in the starter and grower phases compared to the RPC group.

## 4. Discussion

In this study, a standard protein diet with 85% TSAA SP2 showed improved growth performance compared to the standard protein diet with 100% TSAA SP1. RPC cysteine supplementation in the reduced protein diet hampered the negative effect of the reduced protein diet and improved growth performance. Glycine supplementation in the diet also inhibited the negative effects of reduced protein, and RPGC also uplifted cysteine aptitude and resulted in similar growth performance as SP2. The acceptable growth performance in this study could also be because of an adequate amount of glycine + serine % in a reduced protein diet. Balanced glycine and serine ratio in the reduced protein diet also improved total sulfur amino acids balance [14]. Moreover, glycine improved nutrient utilization and protein synthesis, thus improving growth performance [23]. This statement supports our judgment that glycine supplementation in a reduced protein diet reduced the negative effect on growth performance, which is also in agreement with Wang et al., 2020 [24]. Also, L-methionine could also be a reason for acceptable growth performance. L-methionine has higher bioavailability for feed efficiency, thus improving growth performance [25].

Reduced protein diet affected blood biochemical parameters; however, the fluctuations were within physiological ranges [26]. TP and ALB are the main transport proteins and it represent the nutritional condition of the chicken [27]. TP and ALB level depends on the AA intake in the diet. The higher level of ALB could also be associated with synthetic amino acid supplementation in the diet [28]. UA concentrations are the indicator of amino acid utilization in chickens fed amino acid-deficient diets [29] and CRE is a product of creatine phosphate in muscle tissue, and its production is proportional to muscle mass [30]. The higher glucose level by a reduced protein diet could be because of the increased intake of nutrients and decrease insulin sensitivity affecting the transport and utilization of GLU [31]. This judgement is supported by increased ADFI in the grower phase by broiler chickens fed on the RPGC diet.

The higher proportion of free amino acids was observed in the broiler chickens fed a reduced protein diet. This could be because broiler chickens utilize amino acids more efficiently, as these amino acids are involved in protein synthesis and metabolic processes. Exemplary growth performance by SP2 is the evidence for our statement. The increased concentrations of taurine, phosphoethanolamine, threonine, valine, isoleucine, phenylalanine, ornithine, and lysine by RPC, and glutamate, glycine, and cysteine by RPG could be because, free amino acids are considered to be completely digestible or fewer amino acids used for protein synthesis and other metabolic processes because nutrient required for protein synthesis was limited [19]. And restricted growth in broiler chicken fed reduced protein diets could be because of the lower availability of amino acids. Lower concentrations of nonessential amino acids reduced the growth of broiler chickens [19].

Serum metabolites showed that SP2 up-regulated the Dihydro-4,4-dimethyl-2,3-furandione, Lys-Asn, and Glycerol 1-myristate. RPGC up-regulated the Benzamide, Pro-Ser, N-Carbamylglutamate, D-gluconate, and Gamma-Glutamylcysteine; however, it downregulated the Dihydro-4,4-dimethyl-2,3-furandione, Ser-His, Lys-Asn Glycerol, and 1-myristate. These results demonstrated that glycine supplementation restricts the adverse effect of a reduced protein diet and uplifted the many processes to accomplished acceptable growth performance.

A reduced protein diet with or without supplementation did not affect carcass characteristics except abdominal fat percentage. All reduced protein diet groups resulted in higher abdominal fat percentage compared to the SP2 group. This is in agreement with references [31,32,33], who reported increased abdominal fat caused by the reduced protein diet. Increased abdominal fat percentage in our experiment could be because of reduced protein and deficiency of methionine in the diet. The reduced protein diet promotes fatty acid synthesis [31]; thus, the methionine-deficient diet increased abdominal fat accumulation [34,35]. The reduced protein diet changed the lipid metabolism and improved abdominal fat deposition [31]. However, glycine supplementation in the reduced protein diet non-significantly reduced the abdominal fat percentage.

In our study, the broiler chickens fed the RP diet significantly reduced their nitrogen excretion compared to the broiler chickens fed a standard protein diet. The nitrogen digestibility was higher in the RP and RPC diets. The reduced protein diet increased nitrogen utilization [36]. Each percent reduction of protein in the diet reduced 10% of the N excretion [36]. Our study confirmed that a reduced protein diet reduced nitrogen excretion, which is in agreement with the reduction of nitrogen emissions in feces [19,31,32,36,37,38].

## 5. Conclusions

Our experiment confirmed that protein could be reduced by 4.5% with optimal supplementation of glycine and cysteine in the corn–soybean meal-based broiler chicken’s diet. A balance between methionine and cysteine with 0.1% cysteine and around 1% glycine in reduced protein diet is the preferable level for better growth in broiler chickens. Moreover, serum metabolomics showed that supplementation of 0.1% cysteine and 1% glycine in reduced protein diet up-regulated the metabolic processes and thus promoted growth performance.

## Figures and Tables

**Table 1:** Dietary composition of standard and reduced protein diets.

Ingredients	Starter Phase (0~21 d)			Grower Phase (21~42 d)		
	SP1	SP2	RP	SP1	SP2	RP
Corn	55.71	55.837	69.59	60.21	60.33	74.698
Soybean meal	35.38	35.38	20.62	30.3	30.3	14.27
Vegetable oil	4.43	4.43	2.67	5.22	5.22	3.33
Dicalcium phosphate	2.27	2.27	2.35	1.72	1.72	1.81
Limestone	0.98	0.98	1.08	1.1	1.1	1.18
Salt	0.16	0.16	0	0.16	0.16	0
L-methionine (99%)	0.25	0.123	0.24	0.25	0.13	0.25
Lysine HCL (78%)	0.12	0.12	0.57	0.15	0.15	0.63
Threonine (98%)	0.05	0.05	0.25	0.07	0.07	0.29
L-arginine (99%)	0	0	0.32	0	0	0.47
L-valine (99%)	0	0	0.21	0	0	0.27
L-isoleucine (98%)	0	0	0.2	0	0	0.24
L-tryptophan (99%)	0	0	0.05	0	0	0.07
L-phenylalanine (99%)	0	0	0	0	0	0.12
L-leucine (99%)	0	0	0	0	0	0.08
Vitamin premix ^1^	0.02	0.02	0.02	0.02	0.02	0.02
Mineral premix ^2^	0.2	0.2	0.2	0.2	0.2	0.2
Choline chloride (50%)	0.1	0.1	0.1	0.1	0.1	0.1
Na and K bicarbonate mix ^3^	0.33	0.33	0.5	0.5	0.5	0.7
Carrier	0	0	1.03	0	0	1.272
Inclusion level	100	100	100	100	100	100

^1^ The vitamin premix supplied the following per kg of complete feed: vitamin A, 12,500 IU; vitamin D3, 2500 IU; vitamin K3, 2.65 mg; vitamin B1, 2 mg; vitamin B2, 6 mg; vitamin B12, 0.025 mg; vitamin E, 50 IU; biotin, 0.0325 mg; folic acid, 1.25 mg; pantothenic acid, 12 mg; niacin, 50 mg. ^2^ The mineral premix supplied the following per kg of complete feed: Cu, 8 mg; Zn, 75 mg; Fe, 80 mg; Mn, 100 mg; Se, 0.15 mg; I, 0.35 mg. ^3^ Contains 66.67% sodium bicarbonate and 33.33% potassium bicarbonate. SP1 = standard protein diet with 100% TSAA; SP2 = Standard Protein diet with 85% TSAA; RP: reduced protein diet; TSAA = total sulfur amino acids.

**Table 2:** Nutritional level of standard and reduced protein diets.

Nutrients Level ^1^	Starter Phase (0~21 d)			Grower Phase (21~42 d)		
	SP1	SP2	RP	SP1	SP2	RP
CP (%)	22 (22.33)	22 (22.30)	17.5 (17.42)	20 (20.24)	20 (20.31)	15.5 (15.46)
ME (Kcal/kg)	3000	3000	3000	3100	3100	3100
TD Lysine	1.12	1.12	1.12	1.12	1.12	1.12
TD TSAA	0.81	0.81	0.81	0.81	0.81	0.81
TD Threonine	0.75	0.75	0.75	0.75	0.75	0.75
TD Tryptophan	0.24	0.24	0.18	0.24	0.24	0.18
Lysine (%)	1.15 (1.28)	1.15 (1.27)	1.15 (1.26)	1.05 (1.19)	1.05 (1.17)	1.05 (1.18)
TSAA (%)	0.83 (0.88)	0.70 (0.75)	0.70 (0.76)	0.79 (0.84)	0.68 (0.75)	0.68 (0.74)
Methionine (%)	0.54 (0.59)	0.42 (0.47)	0.47 (0.53)	0.52 (0.58)	0.40 (0.45)	0.39 (0.46)
Threonine (%)	0.75 (0.81)	0.75 (0.80)	0.75 (0.80)	0.69 (0.75)	0.69 (0.75)	0.69 (0.73)
Tryptophan (%)	0.23 (0.26)	0.23 (0.27)	0.21 (0.24)	0.21 (0.25)	0.21 (0.26)	0.18 (0.22)
Valine (%)	0.92 (0.97)	0.92 (0.96)	0.88 (0.93)	0.85 (0.89)	0.85 (0.87)	0.84 (0.88)
Arginine (%)	1.34 (1.41)	1.34 (1.42)	1.24 (1.30)	1.17 (1.28)	1.17 (1.29)	1.17 (1.26)
Isoleucine (%)	0.84 (0.88)	0.84 (0.87)	0.82 (0.84)	0.75 (0.81)	0.75 (0.79)	0.75 (0.78)
Leucine (%)	1.67 (1.77)	1.67 (1.75)	1.32 (1.43)	1.49 (1.56)	1.49 (1.57)	1.13 (1.26)
Histidine (%)	0.54 (0.58)	0.54 (0.58)	0.41 (0.43)	0.48 (0.54)	0.48 (0.54)	0.41 (0.45)
Phenylalanine + tyrosine (%)	1.63 (1.78)	1.63 (1.76)	1.33 (1.45)	1.54 (1.68)	1.54 (1.70)	1.22 (1.39)
Glycine + serine (%)	1.95 (2.06)	1.95 (2.07)	1.39 (1.51)	1.80 (1.88)	1.80 (1.86)	1.17 (1.23)

^1^ TD = true ileal digestible. Values in the parentheses were analyzed value. SP1 = Standard Protein diet with 100% TSAA; SP2 = standard protein diet with 85% TSAA; RP: reduced protein diet; TSAA = total sulfur amino acids. CP = crude protein; ME = metabolizable energy.

**Table 3:** Sulfur-containing amino acids and glycine addition levels in the reduced protein diets.

RPs	Cysteine		1% Glycine + Cysteine	
	RP	RPC	RPG	RPGC
**Starter phase (0~21 d)**				
L-methionine (%)	0.24	0.14	0.24	0.14
^1^ L-cysteine HCl H_2_O (%)	0	0.147	0	0.147
Glycine (99%) (%)	0	0	0.936	0.936
Carrier (%)	1.03	0.983	0.094	0.047
**Grower phase (21~42 d)**				
L-methionine (%)	0.25	0.15	0.25	0.15
^1^ L-cysteine HCl H_2_O (%)	0	0.146	0	0.146
Glycine (99%)	0	0	1.18	1.18
Carrier (%)	1.272	1.226	0.092	0.046

^1^ L-cysteine HCl H_2_O contained 68.3% L-Cysteine. RP = reduced protein diet; RPC = reduced protein diet with cysteine supplementation; RPG = reduced protein diet with glycine supplementation; RPGC = reduced protein diet with cysteine and glycine supplementation; L-methionine = Levo methionine; L-cysteine HCl H_2_O = Levo cysteine hydrochloride monohydrate.

**Table 4:** Effect of standard or reduced protein diets (means ± standard errors) on growth performance of broiler chickens.

Treatments	Starter Phase (0~21 d)				Grower Phase (21~42 d)				Entire Feeding Period (0~42 d)		
	BW (g)	ADG (g)	ADFI (g)	FCR	BW (g)	ADG (g)	ADFI (g)	FCR	ADG (g)	ADFI (g)	FCR
SP1 (*n* = 72)	934.9 ± 13.8	41.5 ± 0.64	51.8 ± 0.83	1.25 ± 0.0	2649.6 ± 70.4	76.5 ± 2.52	128.2 ± 2.04	1.68 ± 0.05	58.4 ± 1.33	88.6 ± 1.14	1.52 ± 0.03
SP2 (*n* = 72)	970.6 ± 13.8	43.4 ± 0.69	54.3 ± 0.81	1.25 ± 0.01	2835.8 ± 68.7	84.2 ± 3.13	139.1 ± 4.08	1.65 ± 0.02	63.1 ± 1.73	95.3 ± 2.36	1.51 ± 0.01
RP (*n* = 72)	707.9 ± 13.0	30.7 ± 0.44	43.2 ± 0.63	1.40 ± 0.01	2059.3 ± 25.2	59.0 ± 0.50	110.8 ± 0.98	1.88 ± 0.02	44.3 ± 0.35	75.5 ± 0.69	1.71 ± 0.01
RPC (*n* = 72)	887.3 ± 18.3	37.9 ± 1.01	51.6 ± 1.41	1.36 ± 0.01	2660.9 ± 71.1	76.8 ± 2.84	136.8 ± 3.62	1.78 ± 0.03	54.4 ± 2.24	87.5 ± 3.55	1.61 ± 0.01
RPG (*n* = 72)	748.4 ± 14.1	32.9 ± 0.78	44.4 ± 0.77	1.35 ± 0.01	2186.0 ± 58.4	65.3 ± 2.24	113.0 ± 2.56	1.74 ± 0.04	48.5 ± 1.55	77.4 ± 1.54	1.60 ± 0.02
RPGC (*n* = 72)	935.7 ± 11.3	41.4 ± 0.80	53.4 ± 1.03	1.29 ± 0.01	2800.7 ± 66.3	84.8 ± 2.60	141.6 ± 4.23	1.67 ± 0.01	62.1 ± 1.77	95.6 ± 2.84	1.54 ± 0.00
***p*-value**											
SP1 vs. SP2	0.017	0.017	0.003	0.004	0.01	0.015	0.036	0.023	<0.001	<0.001	<0.001
SP2 vs. RPs	<0.001	<0.001	<0.001	<0.001	<0.001	<0.001	<0.001	<0.001	<0.001	<0.001	<0.001
RP vs. RPC	<0.001	0.001	0.001	0.003	<0.001	0.002	0.002	0.039	<0.001	<0.001	0.002
RP vs. RPG	0.022	0.014	0.018	0.003	0.006	0.044	0.005	0.033	0.003	0.024	0.001
RPC vs. RPGC	0.051	0.036	0.033	<0.001	0.018	0.042	0.051	0.005	0.012	0.027	<0.001

Note: SP1 = standard protein diet with 100% TSAA; SP2 = standard protein diet with 85% TSAA; RP = reduced protein diet without any supplementation; RPC = reduced protein diet with cysteine supplementation; RPG = reduced protein diet with glycine supplementation; RPGC = reduced protein diet with cysteine and glycine supplementation; RPs = RP, RPC, RPG, and RPGC; BW = body weight; ADG = average daily gain; ADFI = average daily feed intake; FCR = Ffeed conversion ratio (feed: gain, g:g).

**Table 5:** Effect of standard or reduced protein diets (means ± standard errors) on blood biochemical parameters of broiler chickens.

Items	TP g/L	ALB g/L	UA mmol/L	BUN mmol/L	GLU mmol/L	CRE mmol/L	ALT U/L	AST U/L
**Starter phase (0~21 d)**								
SP1 (*n* = 6)	31.7 ± 0.65	15.48 ± 0.70	192.63 ± 1.84	0.85 ± 0.02	9.38 ± 0.30	22.80 ± 0.60	13.52 ± 0.81	117.67 ± 6.92
SP2 (*n* = 6)	32.99 ± 0.74	15.79 ± 0.56	184.33 ± 5.36	0.79 ± 0.04	8.80 ± 0.31	22.88 ± 1.70	12.75 ± 1.25	118.05 ± 4.62
RP (*n* = 6)	31.17 ± 0.84	15.01 ± 0.76	192.73 ± 9.55	0.94 ± 0.03	10.66 ± 0.54	21.16 ± 1.88	11.25 ± 0.73	130.25 ± 5.56
RPC (*n* = 6)	32.29 ± 0.64	14.71 ± 0.54	178.16 ± 7.02	0.88 ± 0.04	9.86 ± 0.42	22.26 ± 0.51	12.04 ± 0.84	129.29 ± 5.84
RPG (*n* = 6)	30.43 ± 0.76	14.64 ± 0.40	193.15 ± 5.77	0.86 ± 0.04	10.03 ± 0.34	22.51 ± 0.87	11.99 ± 1.16	130.63 ± 4.02
RPGC (*n* = 6)	32.44 ± 0.67	14.67 ± 0.47	192.57 ± 9.53	0.86 ± 0.05	10.50 ± 1.01	19.05 ± 1.26	13.62 ± 0.93	130.61 ± 4.30
*p*-value								
SP1 vs. SP2	0.034	0.028	0.013	0.034	0.017	0.027	0.036	0.024
SP2 vs. RPs	0.097	0.570	0.557	0.205	0.213	0.287	0.534	0.323
RP vs. RPC	0.031	0.046	0.037	0.036	0.020	0.047	0.059	0.010
RP vs. RPG	0.046	0.018	0.037	0.017	0.019	0.051	0.017	0.051
RPC vs. RPGC	0.018	0.020	0.035	0.018	0.036	0.028	0.023	0.019
**Grower phase (21~42 d)**								
SP1 (*n* = 6)	30.23 ± 0.62	14.82 ± 0.41	153.71 ± 7.30	0.82 ± 0.03	9.01 ± 0.47	22.19 ± 1.15	11.91 ± 0.71	118.66 ± 4.78
SP2 (*n* = 6)	30.76 ± 0.90	14.47 ± 0.44	146.04 ± 5.26	0.79 ± 0.03	9.66 ± 0.10	22.72 ± 2.08	10.65 ± 0.25	130.18 ± 5.84
RP (*n* = 6)	32.31 ± 1.22	14.49 ± 0.35	134.12 ± 4.28	0.81 ± 0.02	9.97 ± 0.72	19.95 ± 2.13	12.36 ± 0.88	124.28 ± 5.52
RPC (*n* = 6)	31.39 ± 0.30	14.02 ± 0.36	138.52 ± 4.59	0.86 ± 0.04	9.23 ± 0.65	25.78 ± 1.83	14.21 ± 1.07	120.98 ± 5.62
RPG (*n* = 6)	31.09 ± 0.87	14.64 ± 0.75	149.49 ± 3.63	0.94 ± 0.06	10.53 ± 0.22	23.09 ± 1.56	12.21 ± 0.85	123.90 ± 6.47
RPGC (*n* = 6)	32.07 ± 0.67	15.59 ± 0.37	147.65 ± 11.14	0.87 ± 0.04	10.88 ± 0.18	20.69 ± 0.79	12.79 ± 0.80	120.77 ± 5.69
*p*-value								
SP1 vs. SP2	0.026	0.013	0.023	0.372	0.026	0.007	0.019	0.026
SP2 vs. RPs	0.669	0.069	0.401	0.076	0.108	0.175	0.073	0.789
RP vs. RPC	0.057	0.027	0.027	0.346	0.050	0.010	0.026	0.029
RP vs. RPG	0.044	0.042	0.016	0.123	0.026	0.013	0.034	0.014
RPC vs. RPGC	0.042	0.020	0.046	0.113	0.051	0.023	0.044	0.024

Note: SP1 = standard protein diet with 100% TSAA; SP2 = standard protein diet with 85% TSAA; RP = reduced protein diet without any supplementation; RPC = reduced protein diet with cysteine supplementation; RPG = reduced protein diet with glycine supplementation; RPGC = reduced protein diet with cysteine and glycine supplementation; RPs = RP, RPC, RPG, and RPGC; TP = total protein; ALB = albumin; UA = uric acid; BUN = blood urea nitrogen; GLU = glucose; CRE = creatinine; ALT = alanine aminotransferase; AST = aspartate aminotransferase.

**Table 6:** Effect of standard or reduced protein diets (means ± standard errors) on free plasma amino acids.

Items (ug/mL)	Phosphoserine	Taurine	Phosphoethanolamine	Urea	Aspartic Acid	Threonine	Serine	Asparagine	Glutamate	Glutamine	Glycine
**Treatments**											
SP1 (*n* = 6)	1.94 ± 0.09	15.43 ± 3.94	0.67 ± 0.06	5.71 ± 0.91	4.17 ± 0.44	51.66 ± 6.64	63.58 ± 6.91	78.36 ± 3.28	26.19 ± 2.03	81.87 ± 4.71	42.60 ± 1.79
SP2 (*n* = 6)	1.72 ± 0.05	8.11 ± 0.71	0.57 ± 0.04	5.86 ± 0.40	3.40 ± 0.24	39.63 ± 3.91	51.84 ± 4.34	56.92 ± 5.61	23.25 ± 1.75	66.70 ± 3.52	32.43 ± 2.11
RP (*n* = 6)	1.86 ± 0.08	16.35 ± 1.62	0.61 ± 0.05 ^b^	6.23 ± 0.91	4.13 ± 0.55	63.58 ± 14.10	37.18 ± 6.62	44.85 ± 12.88	35.10 ± 6.08	61.84 ± 20.48	23.46 ± 2.84
RPC (*n* = 6)	1.94 ± 0.12	21.00 ± 3.65	0.90 ± 0.13	11.72 ± 3.13	5.15 ± 0.6	101.87 ± 5.22	51.57 ± 4.96	57.75 ± 4.59	37.09 ± 2.49	75.09 ± 7.13	32.78 ± 4.13
RPG (*n* = 6)	1.84 ± 0.10 ^a^	12.42 ± 1.04	0.45 ± 0.05	11.42 ± 1.87	4.33 ± 0.81	99.07 ± 9.70	67.82 ± 4.85	43.24 ± 8.17	53.69 ± 4.26	50.10 ± 7.15	143.36 ± 9.14
RPGC (*n* = 6)	1.70 ± 0.05	14.84 ± 1.92	0.85 ± 0.11	13.27 ± 3.93	4.47 ± 0.50	93.19 ± 3.48	88.30 ± 7.38	45.57 ± 4.64	36.40 ± 2.58	51.68 ± 2.65	142.82 ± 13.8
*p*-value											
SP1 vs. SP2	0.05	0.012	0.025	0.892	0.178	0.016	0.019	0.005	0.033	0.029	0.003
SP2 vs. RPs	0.233	0.004	0.008	0.165	0.272	<0.001	<0.001	0.458	<0.001	0.319	<0.001
RP vs. RPC	0.629	0.036	0.019	0.156	0.252	0.023	0.019	0.033	0.015	0.526	0.018
RP vs. RPG	0.848	0.015	0.048	0.037	0.845	0.018	0.006	0.018	0.037	0.063	<0.001
RPC vs. RPGC	0.101	0.016	0.051	0.764	0.406	0.019	0.002	0.019	0.015	0.012	<0.001

Note: SP1 = standard protein diet with 100% TSAA; SP2 = standard protein diet with 85% TSAA; RP = reduced protein diet without any supplementation; RPC = reduced protein diet with cysteine supplementation; RPG = reduced protein diet with glycine supplementation; RPGC = reduced protein diet with cysteine and glycine supplementation; RPs = RP, RPC, RPG, and RPGC.

**Table 7:** Effect of standard or reduced protein diets (means ± standard errors) on free plasma amino acids.

Items (ug/mL)	Alanine	Citrulline	α Aminobutyric Acid	Valine	Cysteine	Methionine	Isoleucine	Leucine	Tyrosine	Phenylalanine	β Alanine
**Treatments**											
SP1 (*n* = 6)	86.40 ± 6.29	1.36 ± 0.34	4.12 ± 0.68	18.42 ± 1.23	14.78 ± 0.57	11.93 ± 1.22	11.35 ± 0.78	23.17 ± 1.33	59.26 ± 4.52	22.90 ± 1.50	2.21 ± 0.56
SP2 (*n* = 6)	63.23 ± 4.16	1.42 ± 0.19	2.42 ± 0.14	13.51 ± 0.93	9.45 ± 0.31	4.83 ± 0.30	8.29 ± 0.52	16.92 ± 1.23	45.97 ± 3.63	16.70 ± 1.21	2.43 ± 0.34
RP (*n* = 6)	73.30 ± 11.36	1.36 ± 0.39	4.46 ± 1.02	28.29 ± 2.87	10.85 ± 1.11	12.94 ± 1.67	14.69 ± 1.55	17.91 ± 2.26	40.79 ± 5.67	16.21 ± 0.59	4.03 ± 1.50
RPC (*n* = 6)	76.92 ± 5.96	0.61 ± 0.14	3.69 ± 1.49	34.51 ± 2.36	12.80 ± 0.93	9.59 ± 0.93	17.83 ± 1.87	21.69 ± 1.79	39.28 ± 4.72	22.01 ± 1.13	5.42 ± 1.04
RPG (*n* = 6)	65.93 ± 3.58	1.60 ± 0.24	4.64 ± 0.33	28.24 ± 0.87	13.58 ± 0.79	12.58 ± 0.55	14.55 ± 0.29	16.38 ± 1.29	26.95 ± 2.03	15.78 ± 0.60	3.10 ± 0.50
RPGC (*n* = 6)	64.37 ± 7.15	1.60 ± 0.10	4.10 ± 0.74	27.03 ± 2.48	12.88 ± 0.32	8.21 ± 0.68	13.96 ± 1.60	18.03 ± 1.21	29.92 ± 1.48	20.32 ± 1.18	3.29 ± 0.33
*p*-value											
SP1 vs. SP2	0.013	0.887	0.043	0.01	<0.001	<0.001	0.009	0.006	0.046	0.009	0.748
SP2 vs. RPs	0.530	0.019	0.449	<0.001	0.003	<0.001	0.001	0.166	0.010	0.001	0.130
RP vs. RPC	0.773	0.085	0.689	0.012	0.028	0.019	0.023	0.021	0.048	0.002	0.451
RP vs. RPG	0.554	0.619	0.874	0.005	0.041	0.043	0.029	0.275	0.051	0.012	0.573
RPC vs. RPGC	0.207	<0.001	0.806	0.054	0.036	0.025	0.014	0.012	0.008	0.032	0.079

Note: SP1 = standard protein diet with 100% TSAA; SP2 = standard protein diet with 85% TSAA; RP = reduced protein diet without any supplementation; RPC = reduced protein diet with cysteine supplementation; RPG = reduced protein diet with glycine supplementation; RPGC = reduced protein diet with cysteine and glycine supplementation; RPs = RP, RPC, RPG, and RPGC.

**Table 8:** Effect of standard or reduced protein diets (means ± standard errors) on free plasma amino acids.

Items (ug/mL)	γ Aminobutyric Acid	Histidine	3-Methylhistidine	1-Methylhistidine	Carnosine	Tryptophan	Ornithine	Lysine	Arginine	Hydroxyproline	Proline
**Treatments**											
SP1 (*n* = 6)	2.34 ± 0.64	13.87 ± 1.44	3.68 ± 0.60	5.79 ± 2.94	5.34 ± 1.48	14.11 ± 0.68	4.78 ± 0.71	23.68 ± 5.48	60.81 ± 6.44	22.32 ± 0.82	41.44 ± 3.54
SP2 (*n* = 6)	2.38 ± 0.36	8.07 ± 0.91	2.74 ± 0.50	2.37 ± 0.66	6.64 ± 0.33	12.65 ± 0.78	2.72 ± 0.17	15.18 ± 3.75	63.00 ± 7.03	20.95 ± 1.52	24.59 ± 2.66
RP (*n* = 6)	2.11 ± 0.40	8.42 ± 2.18	1.96 ± 0.26	1.92 ± 0.52	3.69 ± 0.50	13.89 ± 1.28	4.13 ± 0.62	23.86 ± 6.09	77.59 ± 14.77	22.79 ± 4.22	27.15 ± 4.66
RPC (*n* = 6)	1.88 ± 0.17	11.19 ± 2.04	3.27 ± 1.32	3.66 ± 1.42	4.36 ± 0.57	14.52 ± 0.94	8.49 ± 2.22	49.30 ± 9.05	105.59 ± 8.45	22.03 ± 3.05	35.93 ± 2.91
RPG (*n* = 6)	1.75 ± 0.27	10.42 ± 1.19	3.23 ± 0.47	3.59 ± 0.88	4.71 ± 1.56	14.89 ± 0.91	6.92 ± 1.37	33.84 ± 8.70	107.09 ± 9.05	15.98 ± 1.81	29.84 ± 1.80
RPGC (*n* = 6)	2.10 ± 0.31	10.41 ± 0.77	3.38 ± 0.45	2.29 ± 0.44	3.86 ± 0.30	14.28 ± 1.03	6.24 ± 0.82	26.15 ± 4.23	101.78 ± 8.02	13.31 ± 0.68	33.21 ± 2.71
*p*-value											
SP1 vs. SP2	0.96	0.007	0.265	0.314	0.445	0.187	0.024	0.025	0.023	0.042	0.003
SP2 vs. RPs	0.664	0.508	0.679	0.535	0.056	0.551	0.029	0.014	0.009	0.047	0.083
RP vs. RPC	0.579	0.38	0.396	0.315	0.41	0.695	0.011	0.052	0.012	0.008	0.013
RP vs. RPG	0.477	0.444	0.045	0.143	0.548	0.543	0.016	0.037	0.012	0.018	0.05
RPC vs. RPGC	0.546	0.728	0.941	0.379	0.452	0.864	0.036	0.042	0.05	0.019	0.051

Note: SP1 = standard protein diet with 100% TSAA; SP2 = standard protein diet with 85% TSAA; RP = reduced protein diet without any supplementation; RPC = reduced protein diet with cysteine supplementation; RPG = reduced protein diet with glycine supplementation; RPGC = reduced protein diet with cysteine and glycine supplementation; RPs = RP, RPC, RPG, and RPGC.

**Table 9:** Metabolites involved in upregulation and downregulation of the processes of SP2 compared to SP1.

ID	Name	Fold Change	*p*-Value	VIP	Regulated
meta_552	DL-2-Aminoadipic acid	1.866989485	0.048232772	1.613167793	up
meta_595	Dihydro-4,4-dimethyl-2,3-furandione	2.011448525	0.031523444	1.682805005	up
meta_1025	Carvone	0.24803097	0.005133554	2.22179516	down
meta_1463	L-Kynurenine	1.5765205	0.010354845	1.929589769	up
meta_1539	2′-Deoxy-D-ribose	1.567426096	0.013607851	1.923459599	up
meta_2318	Ser-Lys	1.589438202	0.034329294	1.703319241	up
meta_2774	5-methoxytryptophan	1.722896142	0.002150398	2.263481813	up
meta_3787	Ser-Glu	1.533014567	0.046392884	1.645774595	up
meta_4426	Lys-Asn	1.80125319	0.038372499	1.662567759	up
meta_4742	Dimethylbenzimidazole	1.750298124	0.018833095	1.815250214	up
meta_5339	Papaverine	1.720756205	0.00862707	1.926536635	up
meta_5641	Lomefloxacin	1.79005361	0.010637159	1.901273056	up
meta_12121	Glycerol 1-myristate	1.64178141	0.024287119	1.740604498	up

**Table 10:** Metabolites involved in upregulation and downregulation of the processes of RPC compared to RP.

ID	Name	Fold Change	*p*-Value	VIP	Regulated
meta_1654	D-Erythrose 4-phosphate	2.417005252	0.003353	2.519729	up
meta_1778	Adipic acid	1.600202854	0.011132	2.187698	up
meta_3812	Stearidonic Acid	0.437377315	0.044209	1.814022	down
meta_3843	5-Methyl-5,6-Dihydrouracil	1.702955915	0.006437	2.356521	up
meta_4241	Met- Cys	0.663447286	0.047698	1.80482	down
meta_4301	Phthalic acid Mono-2-ethylhexyl Ester	0.649058097	0.022109	1.925922	down
meta_4598	Val-Met	0.260966149	0.004615	2.507538	down
meta_4664	Met-Tyr	0.582431741	0.048361	1.774815	down
meta_4665	N2, N2-Dimethylguanosine	0.619787845	0.037739	1.801952	down
meta_12121	Glycerol 1-myristate	0.337739178	0.000173	2.97313	down

**Table 11:** Metabolites involved in upregulation and downregulation of the processes of RPGC compared to RPC.

ID	Name	Fold Change	*p*-Value	VIP	Regulated
meta_1711	Benzamide	1.510258899	0.021228447	2.105371383	up
meta_1864	Diethyltoluamide	1.570728708	0.000397898	2.848911577	up
meta_2066	Pro-Ser	1.62822336	0.027221486	2.036617136	up
meta_2700	N-Carbamylglutamate	2.47718782	0.00133386	2.899056587	up
meta_2832	D-gluconate	2.479211038	0.004212259	2.699657336	up
meta_2900	L-Anserine	0.468440144	0.049557378	2.022016255	down
meta_3577	Gamma-Glutamylcysteine	1.501156428	0.03613796	2.056078148	up
meta_3642	Lycorine	0.578210523	0.013463678	2.520167579	down
meta_3802	6-Phospho-D-gluconate	1.812509547	0.001027684	2.923297829	up
meta_3987	Ser-His	0.594528194	0.004218219	2.651965493	down
meta_4426	Lys-Asn	0.225256862	0.003887838	2.823747154	down
meta_4598	Val-Met	3.053159658	0.010411407	2.392582108	up
meta_18951	1-Stearoyl-2-oleoyl-sn-glycerol 3-phosphocholine (SOPC)	1.8366737	0.007101341	2.618762561	up

**Table 12:** Metabolites involved in upregulation and downregulation of the processes of RPGC comparison to SP2.

ID	Name	Fold Change	*p*-Value	VIP	Regulated
meta_519	Glutaraldehyde	0.618201927	0.000540002	2.322829384	down
meta_595	Dihydro-4,4-dimethyl-2,3-furandione	0.601671985	0.024467116	1.683088066	down
meta_912	Oxyquinoline	0.580664892	0.001794494	2.334716736	down
meta_932	Coumarin	0.620803735	0.001263471	2.170072721	down
meta_966	(±)-Mevalonolactone	0.634150526	0.007807955	2.001159883	down
meta_1262	Pyruvaldehyde	1.52891377	0.00274563	2.01722229	up
meta_1289	L-Fucose	0.641439889	0.04868351	1.649393212	down
meta_1312	trans-2-Hydroxycinnamic acid	0.614594585	0.000582337	2.3011482	down
meta_1645	L-Tyrosine	0.603893734	0.000764773	2.299952506	down
meta_1711	Benzamide	1.597326918	0.012128092	1.753235171	up
meta_2066	Pro-Ser	1.633845015	0.025050189	1.594178462	up
meta_2402	3-Indoleacetonitrile	0.370148675	2.50E−05	2.599210382	down
meta_2559	Pro-Glu	0.652696159	0.005408547	2.114804635	down
meta_2700	N-Carbamylglutamate	2.467136326	0.001603254	2.178750729	up
meta_2832	D-gluconate	2.886313805	0.002836549	2.091491584	up
meta_3086	Pro-Ala	1.515176238	0.003502953	2.005024211	up
meta_3519	trans-Vaccenic acid	1.90344991	0.024564604	1.613085628	up
meta_3577	Gamma-Glutamylcysteine	1.708785525	0.012266993	1.800310274	up
meta_3645	Imidazoleacetic acid	0.623180165	1.11E−05	2.496207621	down
meta_3683	Chrysin	0.548658461	0.001366239	2.16965064	down
meta_3955	His-Tyr	1.859525729	0.000228103	2.351720538	up
meta_3987	Ser-His	0.53428871	0.000348746	2.383538462	down
meta_4230	Gamma.-L-Glu-.epsilon.-L-Lys	0.629687628	0.035561475	1.770574451	down
meta_4426	Lys-Asn	0.551081252	0.027921123	1.854387228	down
meta_4433	Stearic acid	0.574422348	0.0307923	1.798250172	down
meta_4858	Pro-Tyr	1.51146449	0.028794593	1.584978773	up
meta_7269	25-Hydroxycholesterol	1.846403535	0.033307518	1.458929058	up
meta_7309	1-Stearoyl-rac-glycerol	0.589307897	0.044909219	1.712872839	down
meta_9568	1-Oleoyl-sn-glycero-3-phosphocholine	1.705954216	4.20E−06	2.534077196	up
meta_10685	Retinene	1.572633	0.003404586	1.96221663	up
meta_12121	Glycerol 1-myristate	0.428804023	0.000519244	2.46436168	down
meta_12242	Eicosapentaenoic acid	1.869449926	0.000278104	2.309348832	up
meta_15389	1-Palmitoyl-2-oleoyl-sn-glycero-3-phosphoethanolamine	1.714972169	0.004281934	1.87445855	up

**Table 13:** Effect of standard or reduced protein diets (means ± standard errors) on carcass quality.

Items (%)	Carcass Yield	Breast	Leg	Wing	Abdominal Fat
**Treatments**					
SP1 (*n* = 12)	71.5 ± 1.00	26.1 ± 1.06	31.7 ± 1.15	10.3 ± 0.30	1.72 ± 0.04
SP2 (*n* = 12)	73.4 ± 1.25	25.3 ± 0.41	32.8 ± 1.01	10.0 ± 0.31	1.76 ± 0.17
RP (*n* = 12)	73.6 ± 1.54	26.5 ± 1.71	30.5 ± 0.67	9.77 ± 0.37	2.40 ± 0.20
RPC (*n* = 12)	74.3 ± 0.39	25.9 ± 1.28	31.6 ± 0.55	9.46 ± 0.18	2.53 ± 0.20
RPG (*n* = 12)	73.1 ± 0.78	23.2 ± 0.75	32.3 ± 0.61	10.2 ± 0.22	2.05 ± 0.21
RPGC (*n* = 12)	74.3 ± 0.47	23.6 ± 0.62	32.0 ± 0.46	9.66 ± 0.18	2.57 ± 0.22
*p*-value					
SP1 vs. SP2	0.267	0.492	0.499	0.426	0.032
SP2 vs. RPs	0.882	0.160	0.227	0.326	0.037
RP vs. RPC	0.652	0.801	0.243	0.458	0.036
RP vs. RPG	0.788	0.113	0.081	0.339	0.026
RPC vs. RPGC	0.913	0.131	0.553	0.457	0.038

Note: SP1 = standard protein diet with 100% TSAA; SP2 = standard protein diet with 85% TSAA; RP = reduced protein diet without any supplementation; RPC = reduced protein diet with cysteine supplementation; RPG = reduced protein diet with glycine supplementation; RPGC = reduced protein diet with cysteine and glycine supplementation; RPs = RP, RPC, RPG, and RPGC.

**Table 14:** Effect of standard or reduced protein diets (means ± standard errors) on nitrogen digestibility.

Treatments	Nitrogen Digestibility %	
	Starter Phase (17~19 d)	Grower Phase (27~29 d)
SP1 (*n* = 5)	57.7 ± 4.05	83.1 ± 0.71
SP2 (*n* = 5)	52.8 ± 1.55	81.5 ± 0.43
RP (*n* = 5)	58.2 ± 0.80	87.9 ± 1.22
RPC (*n* = 5)	61.3 ± 1.01	87.6 ± 1.43
RPG (*n* = 5)	56.9 ± 1.23	85.5 ± 0.74
RPGC (*n* = 5)	58.3 ± 1.82	83.5 ± 0.82
*p*-value		
SP1 vs. SP2	0.124	0.044
SP2 vs. RPs	0.006	0.002
RP vs. RPC	0.883	0.039
RP vs. RPG	0.167	0.032
RPC vs. RPGC	0.041	0.018

Note: SP1 = standard protein diet with 100% TSAA; SP2 = standard protein diet with 85% TSAA; RP = reduced protein diet without any supplementation; RPC = reduced protein diet with cysteine supplementation; RPG = reduced protein diet with glycine supplementation; RPGC = reduced protein diet with cysteine and glycine supplementation; RPs = RP, RPC, RPG, and RPGC.
